# Development of Rapid Extraction Method of *Mycobacterium avium* Subspecies *paratuberculosis* DNA from Bovine Stool Samples

**DOI:** 10.3390/diagnostics9020036

**Published:** 2019-03-29

**Authors:** Sören Hansen, Marco Roller, Lamia M. A. Alslim, Susanne Böhlken-Fascher, Kim Fechner, Claus-Peter Czerny, Ahmed Abd El Wahed

**Affiliations:** 1 Division of Microbiology and Animal Hygiene, Department of Animal Sciences, Faculty of Agricultural Sciences, University of Goettingen, D-37077 Goettingen, Germany; soeren.hansen@uni-goettingen.de (S.H.); lamia.alslim@hotmail.com (L.M.A.A.); susanne.boehlken-fascher@agr.uni-goettingen.de (S.B.-F.); kfechne@gwdg.de (K.F.); cczerny@gwdg.de (C.-P.C.); 2 Zoological and Botanical Garden Wilhelma, D-70376 Stuttgart, Germany; marco_roller@web.de

**Keywords:** *Mycobacterium avium* subsp. *paratuberculosis*, rapid extraction, mobile suitcase laboratory, SpeedXtract, point of need extraction

## Abstract

The rapid identification of *Mycobacterium avium* subspecies *paratuberculosis* (MAP) infected animals within the herd is essential for preventing the spread of the disease as well as avoiding human exposure. Although culture is seen as the gold standard, there are various molecular assays available i.e., polymerase chain reaction (PCR) or isothermal amplification technique (recombinase polymerase amplification (RPA)) for the detection of MAP. The accuracy of the molecular assays is highly dependent on the DNA extraction method. In order to establish a rapid point of need system for the detection of MAP DNA from stool samples, we developed a rapid DNA extraction protocol (MAP DNA SpeedXtract) specified for use in combination with the RPA. The whole procedure from “sample in” to “result out” was conducted in a mobile suitcase laboratory. The DNA extraction is based on reverse purification by magnetic beads, which reduces the required technical demand. The MAP DNA SpeedXtract was performed within 25 min and only three pipetting steps were needed. The amplification and detection time were 20 min in RPA. The sensitivity and specificity of the developed protocol in comparison with the lab-based silica membrane column extraction and real-time PCR were 90.9% (*n* = 22) and 100% (*n* = 23), respectively. In conclusion, we established a rapid and reliable protocol for the extraction and detection of MAP DNA. All reagents are cold chain independent. The entire setup is ideal for point of need identification of MAP infected cases.

## 1. Introduction

The *Mycobacterium avium* subspecies *paratuberculosis* (MAP) as the causing agent for Johne’s Disease (Paratuberculosis) in ruminants is a Gram-positive, aerobic, non-motile, non-spore-forming and acid fast bacterium [1]. Clinical signs of John’s disease, such as weight loss, reduction in milk production and progressing diarrhoea, have an enormous negative impact on the dairy industry [2]. Transmission occurs mainly through the faecal–oral route [3]. The identification of subclinical shedders is highly necessary to prevent silent spreading of the pathogen within the herd. Faecal culture is the gold standard for the diagnosis of MAP [4], however it requires at least 12–16 weeks before the sample can be considered as negative. Alternatively, highly sensitive and specific molecular assays such as Polymerase Chain Reaction (PCR) [5] or the recently published Recombinase Polymerase Amplification assay for the detection of MAP (MAP RPA) [6] are available. However, the clinical performance of these assays depends strongly on the quality of the extracted DNA [7]. Isolation of MAP DNA from faecal samples is especially challenging due to the presence of other complex compounds in the bovine faeces, which can inhibit the amplification process. Moreover, the cell walls of MAP, containing high numbers of lipophilic molecules and polysaccharides, are not easy to destroy [8,9,10]. In standard laboratory protocols, physical disruption is applied after adding chaotropic salts and proteinases to the sample. A lab tube containing silica gel membrane columns is used to obtain a highly purified DNA after employing several washing and centrifugation steps [7,11,12]. These procedures are often time consuming, complex and must be conducted at a well-equipped laboratory. In order to provide a diagnostic tool for paratuberculosis at point of need, here we described a rapid extraction protocol (MAP DNA SpeedXtract) based on magnetic bead. The destruction of the MAP cell wall in the SpeedXtract depends on the combination of physical disruption and heat in the presence of a lysis buffer. The magnetic beads capture the cell debris and most contaminants and then leave the nucleic acid free in the supernatant [13,14,15]. Therefore, the SpeedXtract was named a reverse purification method.

## 2. Materials and Methods

### 2.1. Sample Origin

The study included 45 bovine stool samples which were collected during routine veterinary examination in Division of Microbiology and Animal Hygiene, Goettingen. All samples were taken under consideration of the German codex “Gute Veterinärmedizinische Praxis”.

### 2.2. Development of MAP SpeedXtract Protocol

In order to establish a rapid point of need nucleic acid extraction method, 11 different pre-treatment steps (Table 1) were combined with a basic SpeedXtract procedure. All methods were evaluated using bovine faecal sample containing intact MAP particles.

The basic SpeedXtract (QIAgen, Hilden, Germany) was performed as follows: 500 µL of lysis buffer (Buffer SL) and 60 µL of magnetic beads were added to the faecal samples. The mix was vortexed for 10 s and incubated at 95 °C. Every two minutes, the tube was taken out from the heat block and vortexed. Following 15 min of incubation time, the tube was placed on a magnetic rack. After two minutes, 10 µL of the supernatant was diluted in 40 µL nuclease free water.

### 2.3. RPA Assay

The RPA assay was conducted as published previously [6]. Briefly, 5 μL of the diluted supernatant, 29.5 μL Rehydration Buffer, 6.7 μL molecular biology water, 2.1 μL of 10 μM of both Forward Primer (5′-CGTGGACGCCGGTAAGGCCGACCATTACTGCATGG-3′) and Reverse Primer (5′-CGCCGCAATCAACTCCAGCAGCGCGGCCTC-3′), 0.6 μL of the 10 μM of the exo probe (5′-ACGCCGGTAAGGCCGACCATTACTGCATGGT BHQ1-dt, Tetrahydrofuran and Fam-dT TAACGACGACGCGCA-PH-3′) and 2.5 μL of 14 mM Mg acetate were added to a freeze-dried reaction pellet ordered from TwistDx (TwistDx Ltd., Cambridge, UK). The tube was incubated at 42 °C for 15 min. The fluorescence signals were recorded every 30 s. The RPA threshold time was calculated using the first derivative value obtained by the Studio Software (Qiagen Lake Constance, Stockach, Germany).

### 2.4. Clinical Sensitivity and Specificity

The clinical performance of the selected MAP DNA SpeedXtract protocol in combination with the MAP RPA was validated using 100 mg of each of the 45 clinical samples. From the same samples, DNA was extracted using the standard laboratory protocol (QIAamp DNA Blood Mini Kit, (QIAgen GmbH, Hilden, Germany)) and was screened with a well-established IS900 real-time PCR (FP: 5′-TACCGCGGCGAAGGCAAGAC-3′; RP: 5′-CGGAACGTCGGCTGGTCAGG-3′, probe: 5′-FAM-ATGACATCGCAGTCGAGCTG-BHQ-1-3′), as previously described [12].

## 3. Results

**Figure 1:**
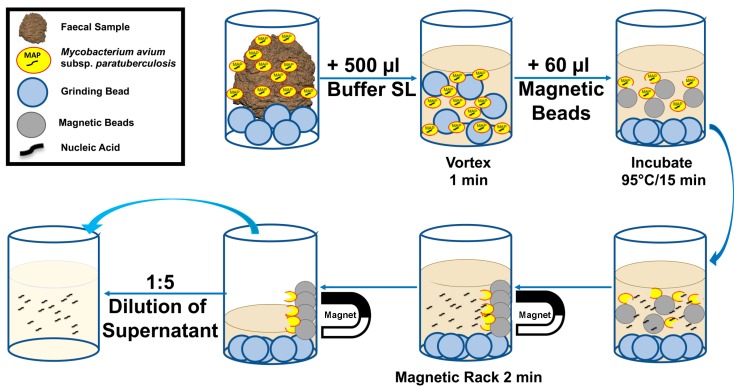
Workflow of the rapid point of need *Mycobacterium avium* subspecies *paratuberculosis* (MAP) extraction protocol. The procedure combines bead beating together with the basic SpeedXtract method. It represents protocol number 10 in Table 1.

**Figure 2:**
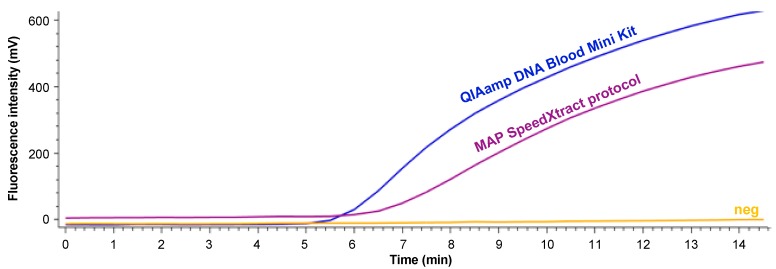
RPA results of DNA extracted either by using the QIAamp DNA Mini Blood Kit (blue) and the MAP SpeedXtract protocol (purple). Neg is negative.

Eleven different pre-treatment steps in combination with a basic SpeedXtract procedure were tested to establish a rapid point of need nucleic acid extraction method. The performance of the extraction protocols was compared with the standard laboratory extraction method using a MAP-positive faecal sample. The results are summarized in Table 1 and Supplementary Figure S1. Protocol #10 was selected as minimal pre-treatment steps and equipment were required (Figure 1), in addition to the production of a comparable result to the standard laboratory procedure (Figure 2). The whole procedure conducted in protocol #10 is illustrated in Figure 1 and Supplementary File S1.

**Figure 3:**
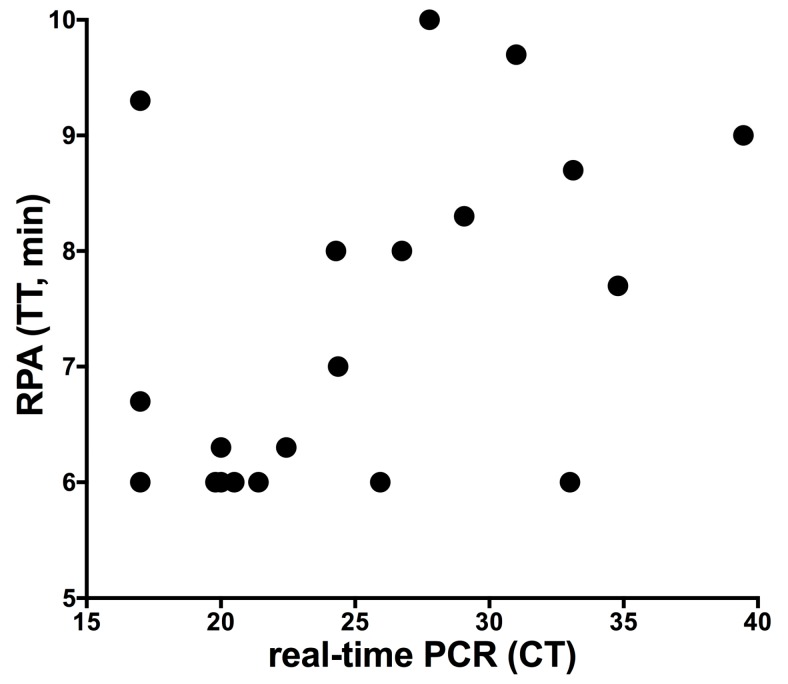
Results of clinical samples screened with both the MAP DNA SpeedXtract/MAP RPA protocol and the QIAamp DNA Blood Mini Kit/real-time Polymerase Chain Reaction (PCR) protocol. No correlation was found as the RPA assay was very fast, even with samples of high CT value. TT is threshold time; CT is cycle threshold.

Each of the 45 faecal samples were mixed well and divided into two parts (100 mg each). DNA was extracted from the first portion with the QIAamp DNA Blood Mini Kit and MAP DNA was detected with real-time PCR, while for the other portion, SpeedXtract and MAP RPA were applied. Comparing the results of both protocols revealed that 23/45 tested samples were negative by both methods. Twenty-two samples tested positive in the real-time PCR, while 20 were positive in the MAP RPA assay. No correlation between the threshold time of the RPA and cycle threshold of the real-time PCR was found (Figure 3).

## 4. Discussion

In this study, we developed a fast and easy to handle MAP DNA extraction and detection method, based on magnetic bead reverse purification and RPA, respectively. The complete procedure was optimized for use in the mobile suitcase laboratory [15]. The protocol reached the same clinical specificity and 90.9 % sensitivity in comparison to the standard laboratory methods.

Many protocols for the extraction of MAP DNA have been developed in the past years (Table 2). All tested methods have showed outstanding clinical sensitivities, however long preparation time and several pipetting steps were necessary. This increases the risk of contamination, especially while working with bovine faecal samples at point of need. Leite et al. applied a rapid MAP extraction procedure [16], nevertheless, a high-speed centrifuge is needed and most centrifuges fail to work under field conditions [17]. Using the SpeedXtract removes the need of a high-speed centrifuge. In addition, the time from sample receiving to result including MAP RPA assay and handling is 45 min and only three pipetting steps are needed. The reverse purification technique, i.e., only inhibitors binding to the magnetic beads, can increase the yield of DNA since no multiple washing and elution steps are required. An additional benefit is that all reagents of the SpeedXtract Nucleic Acid Kit as well as the RPA are stable long term at room temperature, i.e., cold chain independent.

Mondal et al. and Gunaratna et al. applied the basic SpeedXtract Nucleic Acid Kit for the isolation of the *Leishmania donovania* DNA from a blood sample and skin biopsies, respectively [13,15]. Using the SpeedXtract virus kits, Weidmann et al. and Schlottau et al. isolated the Ebola and Rabies viral RNA from blood/swab samples and brain tissue, respectively [18,19]. Here is the first report on deploying the SpeedXtract for bacterial DNA isolation.

MAP colonies from *middlebrook* 7H11 agar plates have a high content of free DNA [20]. Therefore, spiking negative samples with a certain number of bacteria in order to determine the potency of the SpeedXtract was not useful. Thus, we relied on field samples to determine the clinical feasibility of the developed protocol.

The supernatant of the SpeedXtract did inhibit the real-time PCR as its colour stayed dark brown. In other words, the DNA extracted by SpeedXtract is not suitable for any applications including real-time PCR, however this is not the case with the RPA as the RPA is more resistant to an inhibitor than the real-time PCR [6].

The most difficult aspect in the DNA extraction is the lipophilic compounds of the MAP cell wall and clusters which resist acid or alkaline lysis buffers [21]. Bead beating is shown to increase the quality and quantity of yielded DNA [22]. The beads disrupt the cell wall and clusters by causing turbulences and mechanical shearing [23]. Therefore, a bead beating step was implemented in the protocol. As shown in Table 1, implementing treatment with ultrasonic or proteinase or protease gave no additional benefit to the performance in the MAP RPA (Supplementary Figure S1).

## 5. Conclusions

In conclusion, we developed a rapid and sensitive protocol for the extraction of MAP DNA. The uncomplicated setup as well as the minimal technical demand of the SpeedXtract and RPA methods enables implementation in a mobile suitcase laboratory [15]. This eases the detection of MAP shedders within the herd directly at the point of need.

## Figures and Tables

**Table 1:** Different DNA extraction protocols. Eleven variations of the pre-treatment of the sample were applied to select the most field applicable method. Faecal samples were diluted using the Lysis Buffer before pre-treatment step. Magnetic beads were added after the pre-treatment step. TT is Threshold Time of recombinase polymerase amplification (RPA); neg is negative; + is employed in the respective protocol.

Protocol	Pre-Treatment of the Sample							TT (min)	Exponential Curve
	10 min; 40 °C			Ultrasonic (20 kHz, 4 min) (BANDELIN electronic, Berlin, Germany)	Bead Beating (1 min) using Soil Grinding SK38 Precellys Tube (Bertin Corp., Rockville, MD, USA)		SpeedXtract Kit (QIAgen Lake Constance, Stockach, Germany)		
	Sodium Dodecyl Sulfate (20 %) 30 µL (Carl Roth, Karlsruhe, Germany)	Proteinase K 60 µL (Carl Roth, Karlsruhe, Germany)	Protease 5 µL (QIAgen, Hilden, Germany)		on Precellys 24 Tissue Homogenizer (6500 rpm) (Bertin Corp., Rockville, MD, USA)	on Vortex (Scientific Industries, Bohemia, NYC, USA)			
1	+	+		+	+		+	neg	
2		+		+	+		+	6.0	+
3			+	+	+		+	6.0	+
4			+	+			+	neg	
5			+				+	neg	
6				*+*			*+*	neg	
7				+	+		+	6.7	+
8				+		+	+	6.7	
9					+		+	6.3	+
10						+	+	6.7	+
11							+	neg	

**Table 2:** Comparison between different published extraction protocols. + is employed in the respective protocol.

Reference	Kit Used	Kit Producing Company	Purification Method	Time Needed (min)	Sample Amount (mg)	Bead Beating	Heating Step (56 °C–70 °C)	Boiling Step	Proteinase K	Centrifugation	Costs Per reaction (€)
Münster et al., 2013 [11]	QIAmp DNA Blood Mini Kit	Qiagen Hilden, Hilden, Germany	silica gel membrane column	150	100	+	+	+	+	+	5.90
Zang and Zang, 2011 [8]	home-made recipe		silica gel membrane column	160		+	+	+		+	
Leite et al., 2013 [16]	MagMax Total Nucleic Acid Isolation Kit	Applied Biosystems, Foster City, CA, USA	magnetic nucleic acid binding beads	40	300	+				+	5.52
	PowerSoil DNA Isolation Kit	MO BIO Laboratories Inc., Carlsbad, CA, USA	silica gel membrane column	40	300	+	+	+		+	5.00
	QIAamp Stool DNA Mini Kit	Qiagen Hilden, Hilden, Germany	silica gel membrane column	40	1000	+	+	+	+	+	5.78
	ExtractMaster Fecal DNA Extraction Kit	Epicenter Biotechnologies, Madison, WI, USA	inhibitor removal spin column	50	50		+		+	+	unknown
	ZR Fecal DNA MiniPrep	Zymo Research Corp., Irvine, CA, USA	Spin column	20	150	+				+	2.65
	MAP Extraction System	Tetracore Inc., Rockville, MD, USA	Spin column	120	2000	+				+	4.85
Salgado et al., [20]	home-made recipe		centrifugation	160	200	+	+	+	+	+	
MAP DNA SpeedXtract	SpeedXtract Nucleic Acid Kit	Qiagen Lake Constance, Stockach, Germany	inhibitor removal magnetic beads	25	100	+		+			4.75

## Supplementary Materials

The following are available online at https://www.mdpi.com/2075-4418/9/2/36/s1, File S1: MAP SpeedXtract Protocol #10; Figure S1: (A) RPA results of DNA extracted either by the QIAamp DNA Mini Blood Kit (blue) or the MAP SpeedXtract protocol with use of Proteinase K (purple) and without (pink). (B) Performance of the DNA extraction protocols #7 to #10.
