# Supplementary material for: Development of Rapid Extraction Method of Mycobacterium avium Subspecies paratuberculosis DNA from Bovine Stool Samples
Source: Diagnostics (Basel). 2019 Mar 29;9(2):36. doi: 10.3390/diagnostics9020036 (PMC6627389; doi:10.3390/diagnostics9020036)
Supplement: Supplementary file 1 [file diagnostics-09-00036-s001.zip › Supplementary Figure S1.pdf]

Supplementary Figure S1

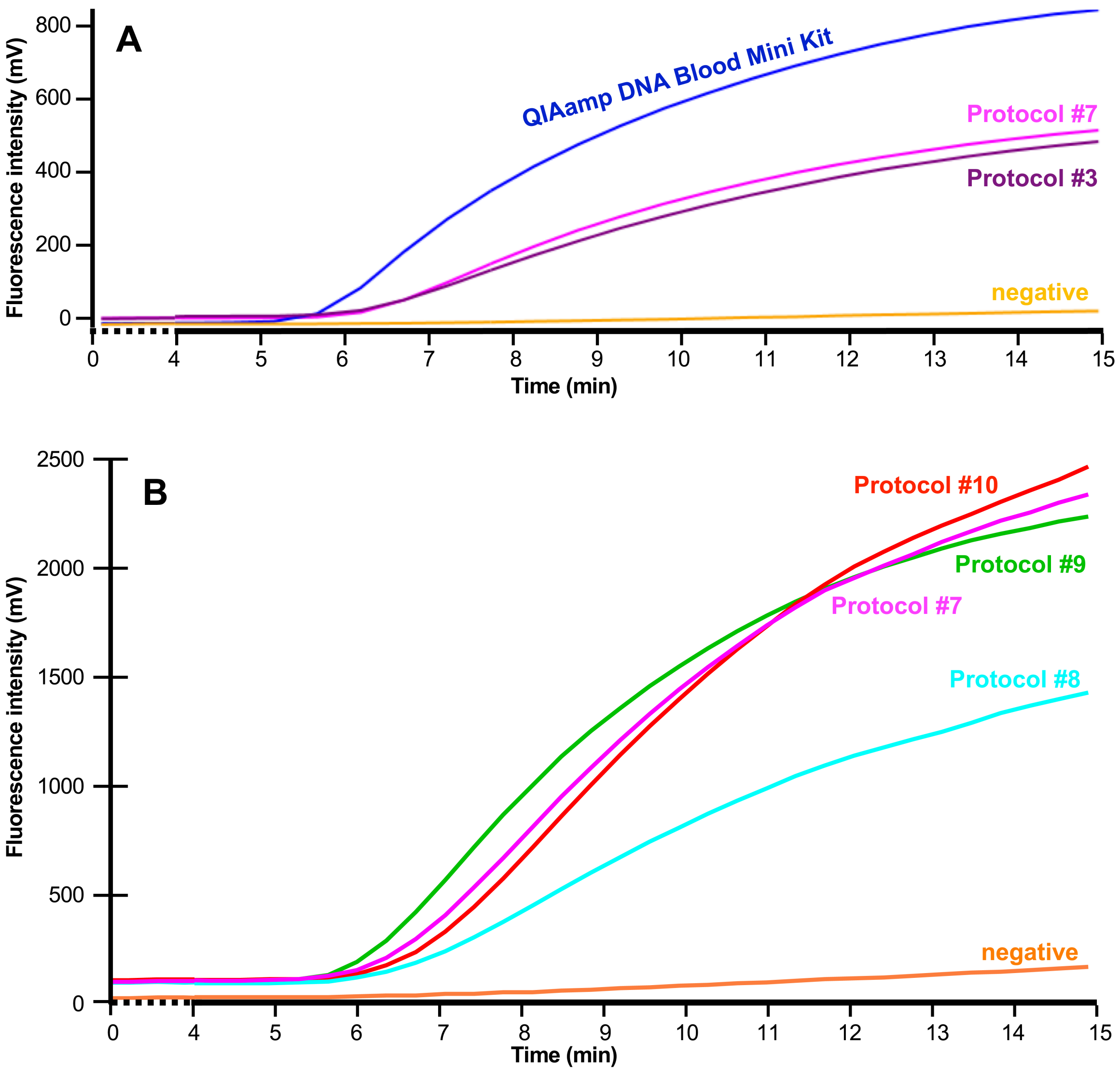

Figure S1. A: RPA results of DNA extracted either by the QIAamp DNA Mini Blood Kit (blue) or the MAP SpeedXtract protocol with use of Proteinase K (purple) and without (pink).
B: Performance of the DNA extraction protocols #7 to #10.
