# Supplementary material for: Development of Rapid Extraction Method of Mycobacterium avium Subspecies paratuberculosis DNA from Bovine Stool Samples
Source: Diagnostics (Basel). 2019 Mar 29;9(2):36. doi: 10.3390/diagnostics9020036 (PMC6627389; doi:10.3390/diagnostics9020036)
Supplement: Supplementary file 1 [file diagnostics-09-00036-s001.zip › Supplementary file 1.pdf]

Supplementary File S1

MAP SpeedXtract Protocol #10

1. Preparations:

- Switch on the heat block and adjust the temperature to 95°C. Please be sure that the heat block reach 95°C before starting with the extraction protocol.
- Prepare for each sample two 1.5 ml tubes containing 40µl water, two Precellys SK 38 tubes and two appropriate tubes for storage. Label them with the sample ID.
- Vortex SpeedXtract Suspension A for 30 seconds to re-suspend magnetic particles.

2. SpeedXtract protocol:

- Add 100 mg of the sample to 500 µl Buffer SL in a Precellys SK 38 tube.
- As an extraction control, please add 500 µl Buffer SL in a Precellys SK 38 tube.
- Place tubes on a Vortex and mix for 1 min
- 60 µl of Suspension A to the sample.
- Mix well for 10 seconds by vortexing or inverting.
- Incubate tubes at 95°C for 15 minutes. Remove the tube every two minutes from the heat block and vortex.
- Remove sample tubes from heat block after 15 minutes and either shake down or tap on a bench to remove condensate from the lid.
- Transfer sample tubes to a magnetic stand and incubate at room temperature for 2 minutes.
- Carefully open sample tubes and transfer 10 µl of the supernatant to a 1.5 ml tube containing 40 µl water to dilute the sample 1:5.
- Put dilute sample direct in RPA and store the rest of the sample at -20°C in an appropriate tube.
